# Equine herpesvirus type 1 (EHV-1)-induced rearrangements of actin filaments in productively infected primary murine neurons

**DOI:** 10.1007/s00705-013-1949-3

**Published:** 2013-12-19

**Authors:** A. Słońska, J. Cymerys, M. M. Godlewski, T. Dzieciątkowski, A. Tucholska, A. Chmielewska, A. Golke, M. W. Bańbura

**Affiliations:** 1 Division of Microbiology, Department of Preclinical Sciences, Faculty of Veterinary Medicine, Warsaw University of Life Sciences, SGGW, Ciszewskiego 8, 02-786 Warsaw, Poland; 2 Division of Physiology, Department of Physiological Sciences, Faculty of Veterinary Medicine, Warsaw University of Life Sciences, SGGW, Nowoursynowska 159, 02-776 Warsaw, Poland; 3 Chair and Department of Medical Microbiology, Medical University of Warsaw, Chałubińskiego 5, 02-004 Warsaw, Poland

## Abstract

Equine herpesvirus type 1 (EHV-1) causes respiratory disease, abortion and neurological disorders in horses. In the present study, we investigated reorganization of the cytoskeleton in neurons infected with two EHV-1 strains: Jan-E (wild-type strain) and Rac-H (attenuated strain). The studies were performed on primary murine neurons, which are an excellent model for studying neurotropism and neurovirulence of EHV-1. We have demonstrated for the first time that EHV-1 infection causes rearrangements in the actin network of neurons that are dependent on the virus strain and its adaptation to cell culture *in vitro*. Immunofluorescent labeling and confocal microscopy revealed the formation of long, thin projections in neurons infected with the Jan-E strain, which was probably associated with enhanced intracellular spread of the virus. The EHV-1 Rac-H strain caused disruption of the microfilaments system and general depolymerization of actin, but treatment of neurons with cytochalasin D or latrunculin A resulted in limitation of viral replication. It can therefore be assumed that actin filaments are required only at the early stages of infection. Our results allow us to suggest that the actin cytoskeleton participates in EHV-1 infection of primary murine neurons but is not essential, and that other components of the cytoskeleton and/or cellular mechanisms may be also involved during EHV-1 infection.

## Introduction

Equine herpesvirus type 1 (EHV-1) is a member of the subfamily *Alphaherpesvirinae* of the family *Herpesviridae.* In its target host, it induces mild respiratory diseases, abortion, neonatal foal death, and neuropathogenic disorders. Like the other α-herpesviruses (HSV-1, BHV-1, PRV), EHV-1 infection is characterized by neurotropism and the ability to establish latent infections in trigeminal ganglion neurons [3, 16, 17, 19].

To initiate effective infection, viruses have evolved a broad spectrum of mechanisms to exploit the host cell, including actin filaments—one of the three major components of the cytoskeleton. The actin cytoskeleton is highly dynamic structure that plays a crucial role in many cellular processes, such as cell division, migration and intracellular transport. Numerous studies have confirmed that various herpesviruses interact with the cell cytoskeleton throughout their life cycle, upon entry, replication or egress, disrupting and rearranging the actin filaments so they can utilize them as tracks or shove them aside when they represent barriers [4, 11, 14, 18, 22, 26].

As a result of established infection, actin polymerization as well as fragmentation of filaments may occur. Infection of neurons by pseudorabies virus (PRV) or herpes simplex virus type 1 (HSV-1) results in formation of actin filaments in the nucleus. Nuclear actin filaments participate in formation of viral capsid assembly sites, while microfilaments serve to transport newly produced virions. This mechanism integrates the viral infectious cycle and the host actin cytoskeleton to promote the infection process [7]. Rearrangement of actin filaments, including elongation and breakdown of fibers, has been observed in fibroblasts during varicella-zoster virus (VZV) infection [12]. Alterations in actin organization in fibroblasts have also been reported for infection with human cytomegalovirus (HCMV), a representative of the β-herpesviruses, which led to disruption of the cytoskeleton and depolymerization of microfilaments [11, 13].

Intracellular transport is critical for the viral replication cycle, particularly for viruses that infect neurons, because they need to pass along a long route from the presynaptic plasma membrane to the nucleus. The virus enters the axon and uses the cytoskeleton for trafficking toward the nucleus, where either the complete replication cycle is initiated or a latent infection is established, or toward the periphery during virus egress [9].

The influence of EHV-1 on the cytoskeleton was previously examined in Vero cells (simian kidney cells) and ED (equine dermal cells) [23, 25]. Reported changes depended not only on the strain of the virus, but also on the origin of the cells. An attenuated strain (Rac-H) disrupted actin fibers and reduced the F-actin level in ED cells, whereas it did not influence the cytoskeleton of Vero cells. A different situation was observed with a wild-type strain (Jan-E), which induced polymerization of F-actin in Vero cells, but not in ED cells [23]. Unfortunately, there is no information available on alterations induced in the cytoskeleton structure of neuronal cells infected with EHV-1, in which the virus is able to establish a latent infection.

In previous studies, we demonstrated that EHV-1 strains replicated in murine neurons *in vitro* without the need for adaptation [1]. We have also examined the mechanism associated with cell death (apoptosis and necrosis) of EHV-1-infected neurons and shown that the majority of infected neurons remained unchanged and survived for more than eight weeks in culture [2]. These results led us to further investigation of the mechanism associated with replication of EHV-1 in murine neurons, in particular with the role of the actin cytoskeleton during intracellular transport of the virus. For that reason, the aim of this study was to investigate the changes induced by different strains of EHV-1 in the morphology and actin network of primary murine neurons.

## Materials and methods

### Nerve cell cultures

A primary culture of murine neurons was established as described before [1]. Cerebral hemispheres were isolated from fetal brains and dissociated in trypsin solution (2.5 %; Gibco) at 37 °C for 15 min. Cells were plated onto poly-D-lysine with laminin or poly-L-lysine- coated cover slips and plates. Neuronal cells were cultured in B-27 neuron plating medium consisting of neurobasal medium, B27 supplement, glutamine (200 mM), glutamate (10 mM), and antibiotics (penicillin and streptomycin), supplemented with 10 % fetal equine serum (Gibco) and maintained at 37 °C with 5 % CO_2_. Four days after plating, the medium was removed and replaced with neuron feeding medium (B-27 neuron plating medium without glutamate). In this medium, murine neurons were maintained for the next 4 days, prior to treatment.

### Virus strains

Cultured primary murine neurons were infected with either of two different strains of EHV-1: strain Jan-E, isolated from an aborted fetus (12^th^ passage in ED cells), or strain Rac-H, which has been passaged through a series of cell cultures and defined as “pantropic, non-pathogenic”. Both strains were obtained from the virus collection of the Virology Laboratory at Warsaw University of Life Sciences–SGGW. The viruses were propagated in equine dermal (ED) and Vero cell cultures grown in Eagle’s minimum essential medium (MEM) (Gibco). Primary murine neuronal cells were infected with approximately 10^5^ TCID_50_/ml of the Jan-E or Rac-H strain of EHV-1. After a one-hour incubation with EHV-1 at an MOI of 0.3, the inoculum was removed and fresh culture medium was added. Subsequently, neuronal cells were incubated for 24, 48 or 72 hours at 37 °C with 5 % CO_2_.

### Immunofluorescent staining procedures

After incubation with the virus, neuronal cells were washed twice in PBS (Sigma Chemicals) and fixed in 3.7 % paraformaldehyde/PBS (Sigma Chemicals) for 10 min at room temperature and suspended in cold acetone (−20 °C) for 5 min. Before staining, fixed neuronal cells were blocked with PBS containing 1 % bovine serum albumin (BSA) (Sigma Chemicals) for 40 min at room temperature. Filament structures of actin were visualised using TRITC-phalloidin conjugate (500 ng/ml; Sigma Chemicals). The presence of viral antigen was detected by direct immunofluorescence, using polyclonal antiserum EHV-1/ERV conjugated to FITC (VMRD, Inc.). Cell nuclei were stained with Bisbenzimidine/Hoechst 33258 according to the manufacturer’s recommendations. Afterwards, cover slips were mounted on microscope slides using anti-fade mounting medium (Sigma Chemicals). Results were evaluated using a confocal microscope (Fluoview FV10i, Olympus) and a scanning cytometer (Scan^R, Olympus).

### Confocal microscopy

Confocal images were acquired using a Fluoview FV10i laser scanning confocal microscope (Olympus Polska Sp. z o.o.) with a 10x air lens and a 60x water-immersion lens, using ultraviolet/visible light LD lasers with excitation at 405 nm, 473 nm and 559 nm to excite Hoechst, FITC and TRITC, respectively. Images were captured using FV10i software (Olympus Polska Sp. z o.o.) and converted to 24-bit tiff files for visualization.

### Scanning cytometry

Triple-stained neuronal cells were analyzed using a Scan^R scanning cytometer (Olympus Polska Sp. z o.o., Warsaw, Poland) with a 40x air lens, using a set of excitation-emission filters: 488 vs. 525 nm, 543 vs. 610 nm and 352 vs. 461 nm for FITC, TRITC and Bisbenzimidine/Hoechst 33258, respectively. Recognition of nuclei was based on DNA-related fluorescence intensity and circularity factor. To separate overlapping nuclei, a system-implemented watershed method of mathematical separation was used. Cell debris and all remaining overlapping nuclei were automatically eliminated from the readout by gating. The fluorescence intensity related to the F-actin and viral antigen was measured in the gated cell population. All experiments were repeated at least five times.

### Treatment with actin inhibitors

Cytochalasin D (CytD, Sigma Chemicals) and latrunculin A (LA, Calbiochem) were used to disrupt actin filaments. Neuronal cells were preincubated with either 1 mM latrunculin A or 10 mM cytochalasin D 30 min before infection and kept in the culture medium during infection to prevent repolymerization of the actin filaments. The appropriate concentrations of actin inhibitors were determined by titration (data not shown). At 24 h p.i., 200 μl of appropriate material was taken for viral DNA isolation, which was performed using a High Pure Viral Nucleic Acid Kit (Roche Diagnostics) according to the manufacturer’s instructions and analyzed using a real-time PCR technique.

### Real-time PCR

The quantity of the EHV-1 DNA in all samples was estimated using real-time PCR (RT-PCR, qPCR) with fluorescent TaqMan probes (150 nM), complementary to a sequence within the amplified products. For amplification of viral DNA, primers (1.75 μM) specific for glycoprotein B (gB) gene were used (gB-1, 5’–AAA CAA AGA GCG GAC CCT AT–3’; gB-2, 5’–TCC GTG AAA ATC TCG TTC TC–3’). Investigations were performed using a TaqMan Master Kit (Roche Diagnostics). Serial dilutions of the Jan-E EHV-1 strain in sterile deionized water in ranges varying from TCID_50_ = 10^6^ (10^6^ copies/ml) to TCID_50_ = 10^2^ (10^2^ copies/ml) were used as reaction standards. Uninfected primary murine neuronal cells were used as a negative control. Each sample was amplified with an internal control (positive control), which consisted of infected neuronal cells incubated without cytoskeleton inhibitors. The qPCR protocol consisted of an activation of termostable hot-start DNA polymerase for 10 min at 95 °C, followed by 40 cycles of denaturation (15 s at 95 °C), primer annealing (20 s at 55 °C) and strand elongation (15 s at 72 °C). Fluorescence levels were read at 560 nm wavelength. Tests were run on a LightCycler 2.0 instrument (Roche Diagnostics) using an in-house quantitative method [5].

### Statistical evaluation

The results were statistically evaluated by one-way analysis of variation (ANOVA) using the Student-Newman-Keuls multiple comparisons test and the Turkey-Kramer multiple comparisons test. These analyses were performed using GraphPad Prism^TM^ version 4.03 software (GraphPad Software Inc., San Diego, CA, USA). Statistical differences were interpreted as significant at *P<0.05*, highly significant at *P<0.01* and not significant at *P>0.05.*

## Results

### Rearrangement of the actin filament organization in EHV-1-infected neurons

**Fig. 1:**
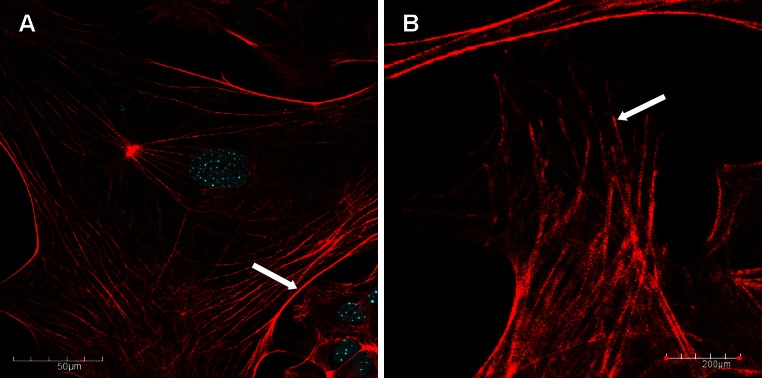
Confocal images of microfilaments (**A**) and the axon growth cone (**B**) in uninfected murine neuronal cells, with staining for actin (*red fluorescence*) and cellular nuclei (*blue fluorescence*). Arrows indicate the regions of actin accumulation

The organization of actin filaments in EHV-1-infected and control neuronal murine cells was examined by confocal microscopy at 24, 48 and 72 h p.i. In control cells, microfilaments form a network of fibers within the cytoplasm; however, the densest distribution was detected in the cortical layer of the cytoplasm in the peripheral region of the plasma membrane (Fig. 1A). Moreover, higher microfilament density was also observed at the synaptic terminals identified as the axon growth cone (Fig. 1B).

**Fig. 2:**
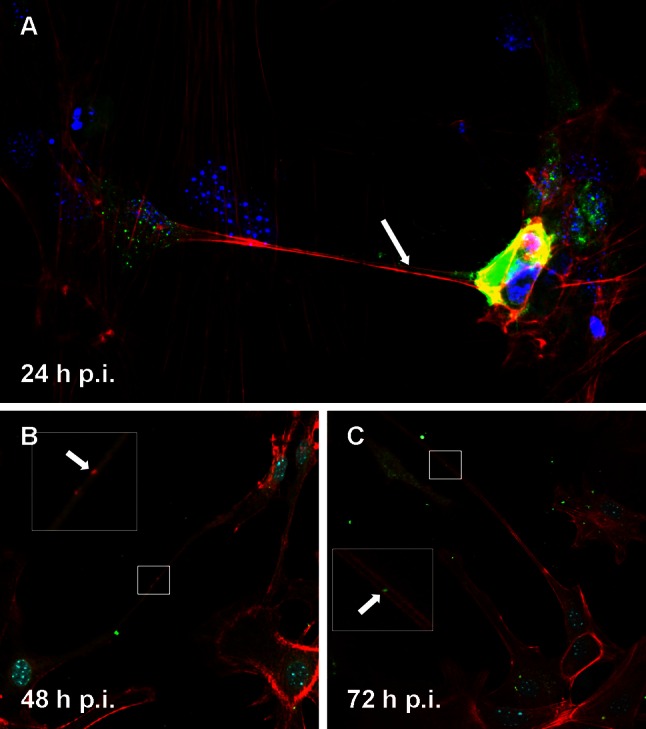
Immunofluorescence image of Jan-E EHV-1-infected neurons, showing viral antigen inside the long, thin actin-rich projections (**A**, **B**, **C**, arrows), with staining for actin (*red fluorescence*), EHV-1 (*green fluorescence*), and cellular nuclei (*blue fluorescence*)

**Fig. 3:**
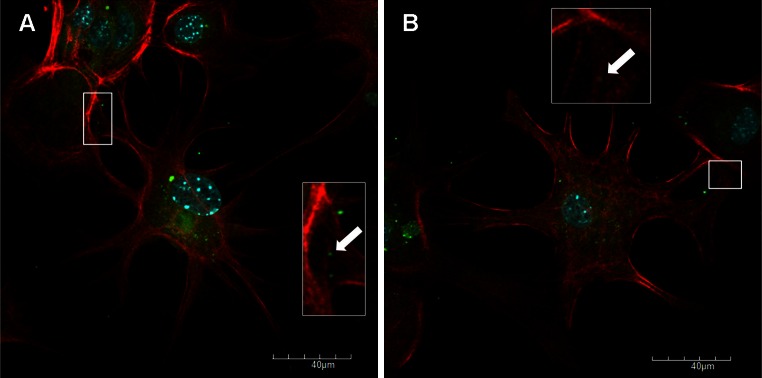
Immunofluorescence image of Jan-E EHV-1-infected neurons (72 h. p.i.), showing virus particles transported inside of short, wide, often branched actin-containing cell projections (**A**, **B**, arrows), with staining for actin (*red fluorescence*), EHV-1 (*green fluorescence*), and cellular nuclei (*blue fluorescence*)

Infection with the Jan-E EHV-1 strain did not disrupt the actin cytoskeleton, but it caused a rearrangement of its distribution. We discovered that the Jan-E strain stimulated actin polymerization in the peripheral region of the neurons and induced formation of long, thin projections (length, 148.96 ± 12.45 μm; width, 1.49 ± 0.25 μm) containing actin fibers that stretched from cell to cell (Fig. 2A). These changes were observed at 24 h p.i., as well as in the subsequent stages of infection (48 and 72 h p.i.) (Fig. 2B, C). Moreover, accumulation of viral antigen inside these long fibers was observed (Fig. 2, arrows). At 48 h p.i., we also observed short, wide, often branched actin-containing cell projections (length, 65.59 ± 8.9 μm; width, 7.38 ± 0.92 μm) that established intimate contact with adjacent cells, and virus particles migrated inside these projections (Fig. 3A, B, arrows). In all cases, the presence of viral antigen in neuronal cells was also detected around the nucleus, which is the site of EHV-1 replication.

**Fig. 4:**
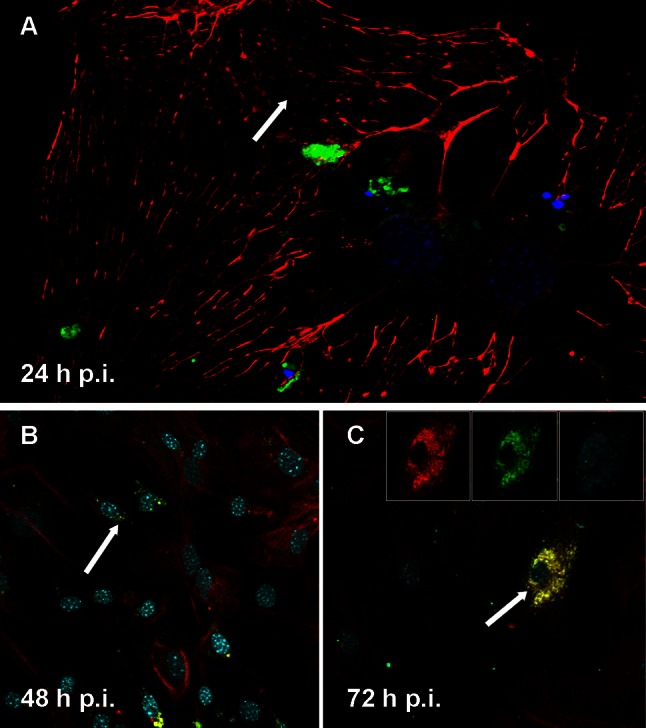
Immunofluorescence image of Rac-H EHV-1-infected neurons, showing disruption of the microfilaments system (**A**, **B**, **C**, arrows), with staining for actin (*red fluorescence*), EHV-1 (*green fluorescence*), and cellular nuclei (*blue fluorescence*)

The Rac-H reference strain of EHV-1 had the opposite effect. As visualized by confocal microscopy, infection with the Rac-H strain led to changes of cell morphology and therefore to changes in the organization of the actin cytoskeleton. Rac-H EHV-1 disrupted the microfilament system and caused general depolymerization of actin (Fig. 4A). These changes increased in the subsequent stages of infection (48 and 72 h p.i.; Fig. 4B, C), when destruction of actin filaments was accompanied by the accumulation of viral antigens and the degradation the nucleus (Fig. 4C).

### Changes in the F-actin level in EHV-1-infected neurons

**Fig. 5:**
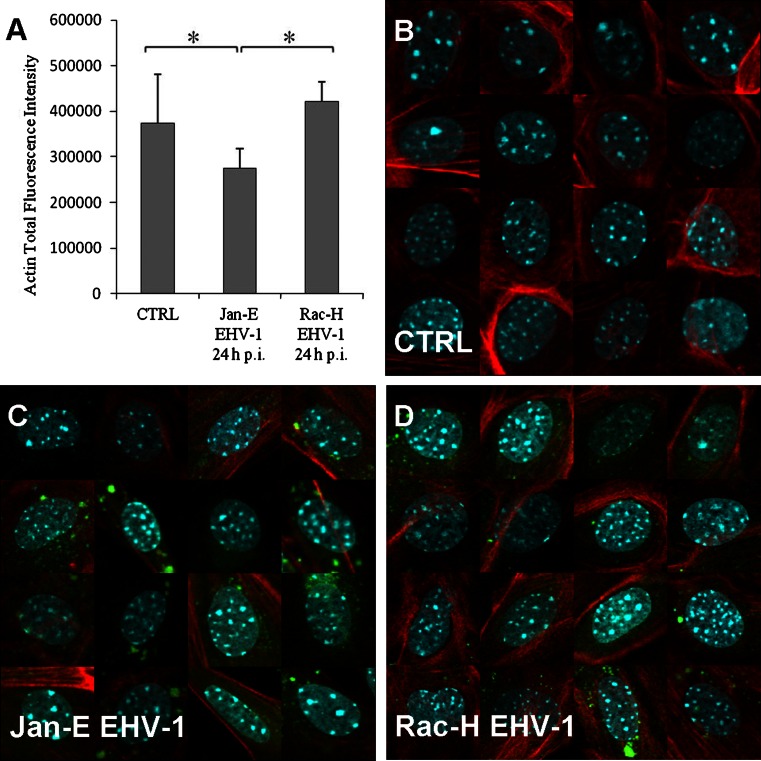
Quantitative evaluation of the F-actin level (**A**) and image gallery of uninfected neurons (**B**), infected with Jan-E EHV-1 (**C**) and Rac-H EHV-1 (**D**), analyzed using the Scan^R scanning cytometry system. A significant decrease in fluorescence intensity of F-actin (*red fluorescence*) in cultures infected with the Jan-E strain compared to control uninfected cells (*, *P<0,05*) was observed, as well as a significant difference in the level of fluorescence intensity between the Jan-E and Rac-H strains (*green* *fluorescence*) (*, *P<0,05*)

To determine whether the observed modifications resulted from actin filament reorganization or from changes in the F-actin protein level, we performed a quantitative evaluation based on Scan^R scanning cytometry, measuring F-actin-related fluorescence intensity. This analysis showed a significant decrease in fluorescence intensity of F-actin in cultures infected with the Jan-E strain (2.75 ± 0.43 × 10^5^; *P<0.05*) compared to control uninfected cells (3.74 ± 1.07 × 10^5^), as well as a significant difference in the level of fluorescence between the Jan-E and Rac-H strains (4.22 ± 0.42 × 10^5^; *P<0.05*) (Fig. 5A). To elucidate these differences, image galleries were created (Fig. 5B-D). As the confocal images clearly demonstrated formation of long thin actin-rich projections induced by the Jan-E EHV-1 strain, the decrease in F-actin fluorescence measured by scanning cytometry was probably not due to destruction of actin filaments but rather to changes in their intracellular localization. These results suggested that EHV-1 could reshape the actin cytoskeleton without changes in F-actin content.

### Effects of the controlled destruction of actin filaments on EHV-1 replication in neurons

**Fig. 6:**
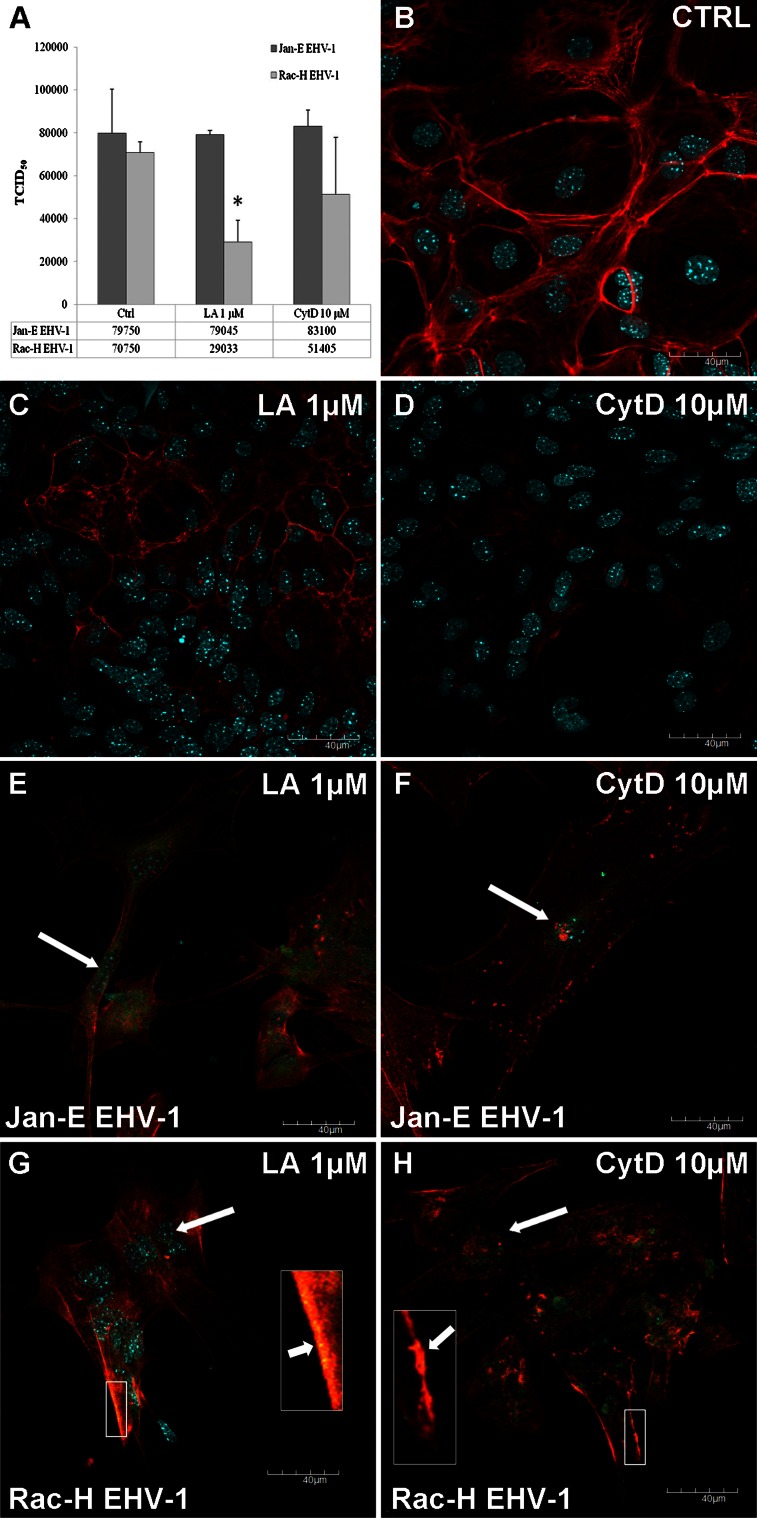
Comparison of viral DNA (TCID_50_) in cells infected with Jan-E or Rac-H EHV-1 (Ctrl) and infected neurons treated with CytD or LA (**A**). A significant difference (*, *P<0.05*) in TCID_50_ was observed between Ctrl and Rac-H EHV-1-infected neurons treated with LA. An immunofluorescence image is shown of uninfected control neurons (Ctrl,** B**), uninfected neurons treated with LA (**C**) and CytD (**D**), Jan-E EHV-1-infected neurons treated with LA (**E**) and CytD (**F**), and Rac-H EHV-1-infected neurons treated with LA (**G**) and CytD (**H**), with staining for actin (*red fluorescence*), EHV-1 (*green fluorescence*), and cellular nuclei (*blue fluorescence*). Large arrows indicate viral antigen around the nucleus; small arrows indicate virions trapped in the cortical cytoplasm

Since our data indicated changes in the organization of cytoskeleton upon EHV-1 infection, we decided to investigate the importance of this filament system by using actin inhibitors—cytochalasin D or latrunculin A—that destabilize actin filaments by inhibiting their polymerization. The effect of inhibitors on neuronal cells was determined using immunofluorescent staining and confocal microscopy. With increasing concentrations of cytochalasin D, visible disappearance of filaments in the cortical cytoplasm and F-actin condensation in the irregularly distributed aggregates was observed (Fig. 6D). After treatment with latrunkulin A, actin filaments were visible mainly in the cortical cytoplasm, but it was clear that their structure was severely altered (Fig. 6C) in comparison with control cells (cells without inhibitor) (Fig. 6B). Despite the apparent destruction of actin filaments by cytochalasin D or latrunculin A, the presence of viral antigen in neuronal cells infected with the Jan-E EHV-1 strain was detected mainly around the nucleus (Fig. 6E, F), whereas in Rac-H EHV-1-infected neurons, the presence of viral antigen was detected around the nucleus, but also in the cortical cytoplasm, where some fragments of actin filaments remained (Fig. 6G, H).

Real-time PCR was applied to detect viral DNA in neuronal cells treated with actin inhibitors in order to determine the level of virus replication (TCID_50_). Treatment of the cells with actin inhibitors (LA and CytD) did not affect the replication of the Jan-E strain (TCID_50_ = 7.90 ± 0.29 × 10^4^ and 8.31 ± 1.04 × 10^4^ for LA and CytD, respectively; *P>0.05*), when compared to control cells without inhibitors (TCID_50_ = 7.97 ± 2.06 × 10^4^). Surprisingly, different results were obtained in neurons infected with Rac-H EHV-1 strain. Depolymerization of actin filaments by treatment with LA (TCID_50_ = 2.90 ± 1.02 × 10^4^; *P<0.05*), as well as CytD (TCID_50_ = 5.14 ± 2.64 × 10^4^; *P>0.05*) limited the replication of the Rac-H strain when compared to control cells without inhibitors (TCID_50_ = 7.07 ± 0.49 × 10^4^). These results suggest that although the replication efficiency was reduced, the inhibitors could not abolish the infection completely (Fig. 6A).

## Discussion

The importance of interactions between viruses and the host cytoskeleton has already been confirmed; however, the mechanism of certain virus-induced cytoskeletal changes and their role during replication still remain unclear, in particular, as regards neurons infected with EHV-1. Although Frampton et al. recently examined the participation of the cytoskeleton in EHV-1 infection, their studies concerned microtubules in non-neuronal heterologous cell lines [8]. Thus, differences between culture models of infection and how cytoskeletal interactions impact infection in animal hosts need to be addressed.

In the present study, we have investigated the impact of two strains – the wild-type EHV-1 strain (Jan-E) and the attenuated EHV-1 strain (Rac-H) – on primary murine neurons. At 24 h p.i., both EHV-1 strains caused different cytopathic effects, indicating that the impact of the virus on neurons may depend on adaptation of the strain to cell culture *in vitro.* These changes were best characterized by abnormal cell morphology, e.g., shrunken, rounded and distorted appearance. Since changes in cell shape are generally related to alterations in the organization of the cytoskeleton, we have decided to investigate possible modulations in the actin network of infected neurons.

Cytoskeleton architecture was observed and evaluated by immunofluorescence and analyzed by confocal microscope and scanning cytometry. At 24 h p.i., the organization of the actin cytoskeleton was altered compared to uninfected control cells. The Jan-E EHV-1 strain caused a rearrangement of the actin distribution and induced the formation of long, thin or short, wide actin-containing cell projections, which stretched from cell to cell and thus may have facilitated virus particles migration (Figs. 2, 3). Above-mentioned structures seemed to contribute to the direct spread of virus particles to adjacent cells without being exposed to the external environment. Moreover, similar projections have been also found to be induced by HSV-1 in Vero and BHK-21 cells and for VZV in HFF cells [6]. For that reason, it can be assumed that this mechanism could be an example of a strategy used by herpesviruses to enhance intercellular spread of infection.

The opposite effect on the actin organization was observed in neurons infected with the Rac-H EHV-1 strain. A significant reduction or even disappearance of actin filaments occurred at 24 h p.i., and this was also observed at the other time points (48 and 72 h p.i.) (Fig. 4). According to other studies, polymerization of actin filaments could serve to propel newly produced virions from cell to cell, while many viruses induce the destruction of selected cytoskeletal filaments, apparently to effect efficient egress [20].

Confocal image analysis has its limitations, because it is based on visualization only, not measurement. In scanning cytometry, the basis for creating the cell gallery is the nucleus. The software is created to show the cell but limited to a certain distance from the nucleus. For neuronal cells, which have numerous projections (dendrites and axons) that can be far away from the nucleus, this distance was insufficient to perform an accurate measurement. For that reason, in the case of the Jan-E strain, at 24 h p.i., when long, thin actin fibres were present at the cell periphery, the loss of F-actin fluorescence was unavoidable. Actin filaments have been found to undergo reorganization but not to decay. Despite these limitations, the confocal images of F-actin in EHV-1-infected neurons confirmed the results obtained by scanning cytometry, whereas the cell gallery served as a verification of these results (Fig. 5).

To determine whether efficient transport of virions through the cytosol depends on an intact cytoskeleton, cells were incubated in the presence of drugs that affect actin filaments. We did not observe any significant perturbations in the level of replication of the Jan-E EHV-1 strain in the presence of cytochalasin D or latrunculin A (Fig. 6A). Thus, when the cytoskeleton was destroyed, the virus was able to employ other transport pathways in the neurons. This hypothesis was confirmed by confocal images in which, despite the destruction of actin filaments, the virus was still able to reach the site of replication (Fig. 6E, F). Moreover, these observations confirmed the results obtained for HSV-1, which showed that depolymerization of actin filaments had no effect on HSV-1 penetration or transport [21].

Unexpected results, however, were obtained for reference strain Rac-H. Even though the virus itself damaged the architecture of the cytoskeleton (24 h p.i.), additional treatment of the neurons with actin inhibitors significantly limited the replication of the Rac-H EHV-1 strain (Fig. 6A). Moreover, confocal images showed that despite the fact that some virions had replicated in the nucleus, some of them were trapped in the cortical cytoplasm, and therefore, their replication cycle was blocked (Fig. 6G, H, arrow). Probably, Rac-H EHV-1 utilized actin filaments at the initial stage of infection, and later, during egress, caused their destruction if they represented a barrier. A similar mechanism has been described for PRV, which was shown to cause transient disassembly of F-actin in swine kidney cells [24]. It is also worth noting that due to the repeated passaging of the Rac-H strain *in vitro,* some parts of the viral genome have been deleted, significantly reducing its virulence. Hübert et al. [10] revealed that this deletion led to the elimination of one or both copies of the gene 67 (IR6) open-reading frame and affected gene 68 (EUS1) in Rac-H. The aggregation of the IR6 to filamentous structures is an important factor that determines EHV-1 virulence and is responsible for its colocalization with type A lamins present in the nucleus. Moreover, the IR6 protein facilitates egress of viral capsids from nuclei of infected cells and enables spread of the viral protein between adjacent cells independently of virus infection [15]. This probably explains the different influence on the actin cytoskeleton between Jan-E and Rac-H EHV-1 strains. Since the F-actin structure was investigated 24 h after infection and later, we can only speculate that the Rac-H EHV-1 strain is dependent on actin filaments during cell entry. In spite of this, the present hypothesis requires verification, and thus the molecular mechanism of reduction of F-actin levels is still being examined in our lab.

Our experiments demonstrated for the first time that EHV-1 infection induces actin cytoskeleton rearrangements, e.g., production of actin-containing profusions and disturbance or even complete degradation of actin fibers, in primary murine neurons. The findings presented here suggest that actin filaments participate, but are not essential, during EHV-1 infection of murine neurons *in vitro*, and even the presence of F-actin destabilizers could not inhibit the infection completely. Our data also revealed that the response of the actin cytoskeleton of murine neurons to infection varied greatly between the EHV-1 strains. Our results may indicate that EHV-1 uses different mechanisms of actin rearrangements during the infection process, where polymerization of actin filaments could serve to propel newly produced virions from cell to cell, whereas the destruction of filaments apparently increases the efficiency of egress. Indeed, the tactics used by the virus to achieve its infectious life cycle are certain to involve multiple interactions with the cytoskeleton. Future studies will aim to define whether other components of the cytoskeleton, e.g., microtubules, motor proteins and intermediate filaments or cellular mechanisms, contribute to a productive infection of neuronal cells. Moreover, knowledge obtained from studies concerning viral intercellular transport and the principles on which it is based may be a new target for antiviral therapy, where the mechanism the drugs will not be based on inhibition of viral enzymes but inhibition of the specific interaction between the virus and the host.
